# Changes of cardiac iron and function during pregnancy in trasfusion-dependent thalassemia patients

**DOI:** 10.1186/1532-429X-18-S1-P270

**Published:** 2016-01-27

**Authors:** Antonella Meloni, Maria Rita Gamberini, Maria Giovanna Neri, Maria Chiara Resta, Gianluca Valeri, Emanuele Grassedonio, Cristina Salvatori, Monica Benni, Antonella Quarta, Vincenzo Positano, Alessia Pepe

**Affiliations:** 1 CMR Unit, Fondazione G. Monasterio CNR-Regione Toscana, Pisa, Italy; 2 Pediatria, Adolescentologia e Talassemia, Arcispedale "S. Anna", Ferrara, Italy; 3 Struttura Complessa di Radiologia, OSP. SS. Annunziata, Taranto, Italy; 4 Dipartimento di Radiologia, Ospedali Riuniti "Umberto I-Lancisi-Salesi", Ancona, Italy; 5 Unità Operativa Sistemi Informatici, Fondazione G. Monasterio CNR-Regione Toscana, Pisa, Italy; 6 Servizio di Immunoematologia e Centro Trasfusionale, Policlinico S. Orsola "L. e A. Seragnoli", Bologna, Italy; 7 Istituto di Radiologia, Policlinico "Paolo Giaccone", Palermo, Italy; 8 Ematologia, Osp. "A. Perrino", Brindisi, Italy

## Background

The aim of this study was to assess the changes in cardiac and hepatic iron overload and in morpho-functional cardiac parameters by Magnetic Resonance Imaging (MRI) in transfusion-dependent thalassemia patients who got pregnant and interrupted their chelation treatment.

## Methods

Among the 956 women with hemoglobinopathies in reproductive age enrolled in the Myocardial Iron Overload in Thalassemia (MIOT) project, we selected 17 women with thalassemia (14 with thalassemia major and 3 with transfusion-dependent thalassemia intermedia) who had a pregnancy with successful delivery and who performed a MRI scan before and after the pregnancy.

Myocardial and liver iron overload were measured by T2* multiecho technique. Atrial dimensions and biventricular function were quantified by cine images

## Results

The pre-pregnancy MRI was performed 15.02 ± 5.31 months before the delivery while the post-partum MRI was performed 5.73 ± 4.45 months later.

For 16 new-mothers the post-partum MRI was performed after the restart of the chelation therapy, specifically 3.95 ± 4.10 months later. One new-mother performed the post-partum MRI about 3 months before restarting the chelation therapy.

The table shows the MRI parameters at the two MRIs.

The pre-pregnancy and the post-partum global heart T2* values and number of pathological segments were comparable. Two patients with a normal global heart T2* value (>20 ms) before pregnancy showed a pathological post-partum value.

After pregnancy there was a significant increase of MRI liver iron concentration (LIC) values. At the pre-partum MRI six (35.3%) patients had a MRI LIC < 3 mg/g/dw while at the post-partum MRI all patients had a pathological MRI LIC.

Among the biventricular volumetric and functional parameters, there was a significant increase of right ventricular (RV) end-systolic volume index and a significant reduction of RV ejection fraction.

## Conclusions

In some transfusion-dependent patients, cessation of chelation therapy allows rapid iron overload. Pregnant women with thalassemia should be monitored carefully for iron loading and cardiac status before they embark upon a pregnancy and afterwards and consideration should be given to offering desferrioxamine chelation therapy immediately after delivery. In women showing severe iron overload before pregnancy desferrioxamine should be started after the middle of the second trimester. The negative impact on the RV parameters could reflect the effect of the high cardiac output state independent of the physiological changes during pregnancy.

**Table 1:** Changes in MRI parameters following the pregnancy.

	Before pregnancy	Post pregnancy	Mean difference	P-value
Global Heart (ms)	33.27 ± 6.72	34.09 ± 9.46	0.82 ± 8.07	0.523
Number of segments with T2* < 20 ms	1.71 ± 2.93	2.35 ± 4.72	0.65 ± 5.44	0.953
MRI LIC (mg/g dw)	4.08 ± 3.55	16.89 ± 8.89	12.82 ± 8.19	<0.0001
LV EDVI (ml/m2)	76.53 ± 8.46	78.53 ± 10.42	2.00 ± 11.95	0.500
LV ESVI (ml/m2)	27.06 ± 3.96	29.24 ± 5.67	2.18 ± 5.37	0.114
LV SVI (ml/m2)	49.41 ± 7.19	47.41 ± 7.28	-2.00 ± 9.69	0.408
LV mass index (g/m2)	51.53 ± 8.43	54.76 ± 9.54	3.24 ± 6.66	0.062
LV EF (%)	64.00 ± 4.64	62.53 ± 4.68	-1.47 ± 5.86	0.317
RV EDVI (ml/m2)	73.24 ± 9.47	75.76 ± 10.94	2.53 ± 11.94	0.395
RV ESVI (ml/m2)	24.24 ± 6.06	27.82 ± 6.44	3.59 ± 6.43	0.035
RV SVI (ml/m2)	47.47 ± 8.35	47.41 ± 7.28	-0.06 ± 10.69	0.982
RV EF (%)	66.82 ± 5.43	63.06 ± 5.51	3.77 ± 5.84	0.017

